# Circulating miRNA’s biomarkers for early detection of hepatocellular carcinoma in Egyptian patients based on machine learning algorithms

**DOI:** 10.1038/s41598-024-54795-2

**Published:** 2024-02-29

**Authors:** Gehad Ismail Sayed, Mona Solyman, Gamalat El Gedawy, Yasmine S. Moemen, Hassan Aboul-Ella, Aboul Ella Hassanien

**Affiliations:** 1 School of Computer Science, Canadian International College (CIC), Cairo, Egypt; 2 Faculty of Computers and Artificial Intelligence, Cairo University, Giza, Egypt; 3 Clinical Biochemistry and Molecular Diagnostics Department, National Liver Institute, Menofia University, Menofia, Egypt; 4 Clinical Pathology Department, National Liver Institute, Menofia University, Menofia, Egypt; 5 Department of Microbiology, Faculty of Veterinary Medicine, Cairo University, Giza, Egypt; 6 College of Business Administration, Kuwait University, Al Shadadiya, Kuwait; 7 Scientific Research Group in Egypt (SRGE), Cairo, Egypt

**Keywords:** Hepatocellular carcinoma, miRNA, Liver cancer, Machine learning, Swarm optimization, Liver cancer, Computational biology and bioinformatics

## Abstract

Liver cancer, which ranks sixth globally and third in cancer-related deaths, is caused by chronic liver disorders and a variety of risk factors. Despite therapeutic improvements, the prognosis for Hepatocellular Carcinoma (HCC) remains poor, with a 5-year survival rate for advanced cases of less than 12%. Although there is a noticeable decrease in the frequency of cases, liver cancer remains a significant worldwide health concern, with estimates surpassing one million cases by 2025. The prevalence of HCC has increased in Egypt, and it includes several neoplasms with distinctive messenger RNA (mRNA) and microRNA (miRNA) expression profiles. In HCC patients, certain miRNAs, such as miRNA-483-5P and miRNA-21, are upregulated, whereas miRNA-155 is elevated in HCV-infected people, encouraging hepatocyte proliferation. Short noncoding RNAs called miRNAs in circulation have the potential as HCC diagnostic and prognostic markers. This paper proposed a model for examining circulating miRNAs as diagnostic and predictive markers for HCC in Egyptian patients and their clinical and pathological characteristics. The proposed HCC detection model consists of three main phases: data preprocessing phase, feature selection based on the proposed Binary African Vulture Optimization Algorithm (BAVO) phase, and finally, classification as well as cross-validation phase. The first phase namely the data preprocessing phase tackle the main problems associated with the adopted datasets. In the feature selection based on the proposed BAVO algorithm phase, a new binary version of the BAVO swarm-based algorithm is introduced to select the relevant markers for HCC. Finally, in the last phase, namely the classification and cross-validation phase, the support vector machine and k-folds cross-validation method are utilized. The proposed model is evaluated on three studies on Egyptians who had HCC. A comparison between the proposed model and traditional statistical studies is reported to demonstrate the superiority of using the machine learning model for evaluating circulating miRNAs as diagnostic markers of HCC. The specificity and sensitivity for differentiation of HCC cases in comparison with the statistical-based method for the first study were 98% against 88% and 99% versus 92%, respectively. The second study revealed the sensitivity and specificity were 97.78% against 90% and 98.89% versus 92.5%, respectively. The third study reported 83.2% against 88.8% and 95.80% versus 92.4%, respectively. Additionally, the results show that circulating miRNA-483-5p, 21, and 155 may be potential new prognostic and early diagnostic biomarkers for HCC.

## Introduction

Liver cancer is the sixth most common malignant tumor in the world and the third greatest cause of cancer-related deaths^1^. Hepatocellular carcinoma (HCC), the most common kind of liver cancer, is caused mostly by chronic liver diseases and multiple variables such as viral infections (hepatitis B and C viruses), alcohol consumption, non-alcoholic fatty liver disease, and genetic mutations^1^. Despite advances in therapeutic techniques such as chemotherapy and immunotherapy, the outlook for HCC remains poor, with a 5-year overall survival rate of less than 12% for advanced cases^2^. Inconspicuous progression in the early stage and metastatic potential in the advanced stage contribute to late diagnosis and poor prognoses^2^. Despite a decline in incidence, liver cancer remains a major worldwide health concern, with a projected incidence of one million cases by 2025^2^. While certain treatments, such as transarterial chemoembolization, are effective for intermediate-stage HCC, dealing with advanced-stage cases remains difficult^2^.

The prevalence of significant risk factors, such as hepatitis C virus (HCV) and hepatitis B virus (HBV) infections, has the greatest influence on HCC incidence. Aflatoxin exposure is a substantial risk factor in Asia and Africa, operating both alone and as a cofactor in chronic HBV infection^3^. Human immunodeficiency virus (HIV) infection in Sub-Saharan Africa increases the risk of HCC in people who have chronic HBV or HCV. Tobacco use is an emerging risk factor, and cirrhosis from diverse sources contributes significantly to the development of HCC. Advanced hepatic fibrosis or cirrhosis is a critical phase in the multistep carcinogenic process in HCV-associated HCC^3^. Alcohol use, coinfection with HBV or HIV, diabetes, older age, race, thrombocytopenia, increased alkaline phosphatase, esophageal varices, and smoking can all increase the risk of HCC in patients with chronic HCV^4^. Recognizing these risk factors permits the identification of high-risk groups as well as early detection through screening, resulting in improved patient outcomes with curative therapies such as liver transplantation, surgical resection, or ablation^3^.

One of the most common malignancies in the world is HCC. Because HCC is typically detected late and there is no curative therapy for an advanced HCC, the death rates for HCC continue to rise by about 2–3% annually, in contrast to the dropping death rates seen for all other prevalent malignancies like breast, lung, and prostate cancers. It is really difficult to diagnose HCC early on. Hope for an early HCC diagnosis is provided by recent technical developments^5^.

While HCC presents substantial challenges for patients, especially given its high mortality rates, it is critical to underline that early detection improves the likelihood of survival significantly. The challenges stem from the aggressive nature of HCC and its propensity for rapid progression. When patients are diagnosed early, their 5-year survival rate exceeds 70%^6^. This highlights the critical role of early identification in improving overall survival outcomes for people dealing with the formidable characteristics of HCC. However, the presence of inflammation and cirrhosis makes early detection of HCC difficult. Therefore, new biomarkers are needed for the early diagnosis of HCC. Currently, imaging methods combined with serum-fetoprotein analysis are used to diagnose HCC without pathological association. Over the past 10 years, improvements in artificial intelligence, and its derived or based techniques, in association with the biomarker assays advancement have led to the discovery of multiple novel biomarkers and better early HCC diagnostic approaches. The most likely candidates for clinical validation in the near future include the most promising biomarkers, such as glypican-3, osteopontin, Golgi protein-73, and nucleic acids, including microRNAs. These biomarkers not only aid in the early detection of HCC but also shed light on the processes behind oncogenesis. Such molecular understanding also lays the groundwork for the development of possibly more efficient treatment regimens^6^.

Early HCC is described as follows in the general rules for the clinical and pathological study of primary liver cancer created by the liver cancer study group of Japan^7^. Focused structural abnormalities in early HCC include acinar or pseudo-glandular formations, broken or crooked trabecular alignment, and/or overt stromal tissue invasion. Normal cellular atypia is unremarkable, but due to reduced cytoplasm, the nuclear-cytoplasmic ratio is elevated. Eosinophilia or basophilia is also visible in the cytoplasm. The cell density may be more than twice as high as the liver tissue around it that is healthy. Lesions frequently show fatty alterations or clear cell abnormalities in addition. Early HCCs have poorly defined margins because the cancer cells do not spread outwardly, but rather multiply by displacing nearby hepatocytes in a trabecular pattern at the edge of the surrounding liver tissues. The lesions are identified macroscopically as tiny nodules with fuzzy borders^8^. These early HCC criteria have previously been accepted internationally^9^ and are included in the most recent "blue book" on digestive system tumors from the world health organization^10^. As a result of the clinical and pathological obstacles that face the early detection of HCC through the conventional clinically established approaches, figuring out an advanced tumor biomarker-based diagnostic technique is the need of the hour to stand against such a serious and widespread tumor.

Oncogenes and tumour suppressor genes are dysregulated during the complicated process of HCC formation and progression. Prior research has shown that microRNA (miRNA) plays a crucial role in oncogenesis by controlling the expression of tumour suppressor and oncogene genes^11^. Non-coding RNA molecules called miRNAs, which have a length of roughly 22 nucleotides, are involved in a number of cellular biological processes, such as cell differentiation and cancer^12^. MiRNAs regulate gene expression at a post-transcriptional level as they bind to specific messenger RNA (mRNA) targets leading to translational repression or mRNA degradation^13,14^. For example, miRNA-21 is an oncogenic miRNA upregulated in many cancers. Through the downregulation of particular tumour suppressor genes, including B-cell lymphoma 2 and tropomyosin 1, phosphatase and tensin homolog, programmed cell death 4 (PDCD4), and apoptosis, cell proliferation, and metastasis are implicated in various malignancy-related processes^15,16^. Asangani et al.^17^ discovered a conserved putative miRNA-21 location inside the 3 untranslated regions of PDCD4 mRNA. They then showed that elevated miRNA-21 controlled PDCD4 levels and caused invasion, intravasation, and metastasis^18,19^. As a result, miRNAs regulate a number of cellular signalling pathways necessary for cell growth, proliferation, motility, and survival^20^. A deeper comprehension of the molecular processes underlying HCC carcinogenesis paves the way for the discovery of novel HCC diagnostic and prognostic biomarkers as well as the identification of possible treatment targets^21–23^.

This paper proposes the use of machine learning (ML) instead of traditional statistical analysis to evaluate circulating miRNAs as diagnostic and prognostic markers for HCC patients and correlate them to their clinical and pathological parameters. Machine learning is a branch of computer science and artificial intelligence that focuses on utilizing data and algorithms to replicate human learning and continuously increase accuracy. Also, it provides a complete analysis of the results for each phase of the proposed model for the three studies. The experimental results prove that the circulating miRNA-483-5p, 21, and 155 could be novel early diagnostic and prognostic biomarkers for detecting HCC. The main contributions of this work can be summarized as follows:

- Machine Learning-based model is proposed to evaluate the diagnostic and prognostic potential (value) of circulating miRNAs in HCC patients and correlating them to the clinical and pathological parameters of the patients using three studies of HCC in Egyptian patients.
- The proposed model provides an early detection model of hepatocellular carcinoma based on miRNA’s biomarkers.
- Solving missing values problem and imbalanced distribution of the clinical report's data for the three studies, which provide a significant improvement in all accuracy measures of the proposed model compared to statistical analysis approaches.
- A new binary version of the African vulture's optimization algorithm based on the lifestyles of African vultures is proposed as a feature selection algorithm.
- Feature selection algorithm indicates a strong relationship between miRNA and the class feature which means that miRNA can be used as prognostic biomarkers for the detection of HCC.

## Materials and methods

An ML-based model is proposed to detect a potential marker for early detection of HCC or a prognostic marker to follow up HCC patients. Three clinical studies were done at the national liver institute (NLI), Menofia University, Menofia, Egypt; these studies targeted HCC patients and chronic liver disease patients and compared them with apparently healthy control persons who have a matched age and gender. In these studies, three different miRNAs were measured (e.g., miRNA-483-5p, miRNA-21, and miRNA-155) in blood to detect a signature of miRNAs in HCC patients. These studies recorded different clinical attributes and parameters besides miRNA related to HCC. A complete description of these parameters and attributes is illustrated in the following section, with a detailed illustration of each study population.

The proposed ML model for evaluating circulating miRNAs as diagnostic markers of HCC consists of three main phases: data pre-processing, feature selection, and finally, classification phase. The pre-processing phase aims to solve problems related to data samples, such as missing values or imbalanced data distribution. Missing data (or missing values) is a data value for a variable in the observation of concern that is not recorded. The missing data issue can greatly impact the inferences you can make from the data^21^. An insertion process for missing values can solve such a problem. One of the most common issues with real-time datasets is data imbalance. If one of the classes in a dataset has a significant dominance over the others, it is said to be unbalanced. Because most ML algorithms for classification were created with the assumption of an equal number of samples for each class, imbalanced classifications make predictive modeling more difficult. Data balancing solutions^22^ have been developed to deal with this situation. Data may be handled in one of two ways: by adjusting current algorithms to give minority classes more weight, so increasing their contribution levels, or by updating the existing dataset to counter unbalancing using sampling approaches^23^. In this research, different sampling methods are employed to solve the problem of unbalanced datasets in the three studies. These methods include the synthetic minority over-sampling technique (SMOTE), the self-adaptive synthetic over-sampling (SASYNO) technique, and the random under-sampling (RUS) method^24^.

The second phase of the proposed model aims to select the best set of features that reflect the identity of each class. It is desirable to reduce the number of input variables to reduce the computational cost of modeling and, in some cases, improve the model's performance. By eliminating unnecessary and duplicated characteristics, feature selection may be thought of as a combinatorial optimization problem that enhances the efficiency of learning algorithms. A novel binary African vulture’s optimization (BAVO) algorithm is proposed to select the most representative features from the input data that can be used later during the classification phase.

In ML, classification is the problem of learning to differentiate samples in a dataset that correspond to two or more classes. To assign patients in each study to their respective classes, the classification phase employs a support vector machine (SVM) as the principal classifier with multiple kernel functions. SVM is a sophisticated approach created through statistical learning.

### Study population

#### The first study with miRNA 483-5p

 The study protocol was performed to evaluate serum miRNA-483-5p quantity in three groups; 50 HCC patients, 25 liver cirrhosis patients, and 25 healthy controls. These evaluations were based on biochemical, coagulation, and hematological assessment for the three included groups. Besides comparing serum miRNA levels before and after the surgical resection of HCC, data processing, and statistical analysis were described before^25^.

#### The second study with miRNA-21

 This study included 30 newly diagnosed cases (26 males and four females) with HCC (30–52 years) and 20 HCV positive cases; Chronic Liver Disease (CLD) patients (15 males and five females) aged from 35 to 51 years selected from inpatient wards and outpatient clinics, national liver institute, Menofia University from January 2014 to December 2014. In addition, 20 healthy people (18 males and two females) with similar ages and gender served as the control group. Their ages ranged from 32 to 53 years. Laboratory tests, clinical examination, ultrasonography, and spiral Computed Tomography (CT) were used to diagnose HCC in the HCC diagnosis group. In addition to biochemical evidence of parenchymal damage, liver biopsy and ultrasonographic features (such as a shrunken liver, a coarse echo pattern, an attenuated hepatic vein, and a fine nodular surface) were used to identify patients with CLD. Complete clinical examinations, abdominal ultrasonography, and/or CT scans were performed on all patients and control groups. Molecular testing, miRNA extraction, cDNA synthesis, amplification, relative quantification, statistical analysis, results, and discussion, were mentioned in a former research paper^26^.

#### The third study with miRNA-155

Included one hundred participants divided into three groups (HCC, HCV, and control groups). All patients were recruited from the National Liver Institute's inpatient wards and outpatient clinics. The patients were divided into the following categories: Group 1 (20 HCC patients with HCV infection): This group consisted of 20 newly diagnosed patients who had not yet started treatment. Clinical examination, laboratory tests, ultrasound, and spiral CT were used to make the diagnosis. Group 2 (60 chronic HCV patients): This group includes 20 chronic HCV patients who did not receive HCV therapy, 20 chronic HCV responded to Interferon treatment (responders), and 20 patients (who were non-responders to treatment to Interferon). Ultrasonography (shrunken liver, coarse echo pattern, attenuated hepatic vein, fine nodular surface) and biochemical indications of parenchymal damage, as well as liver biopsy in some cases, were used to diagnose them. Group 3 (control group) consists of 20 healthy people matching age and gender. The following criteria are for inclusion in this study: 1- triphasic CT or contrast-enhanced dynamic MRI diagnosed HCC. For nodules > 2 cm in diameter in cirrhotic patients, the presence of typical features of arterial enhancement and rapid portal or delayed washout on one imaging technique proved indicative of HCC. Biopsy was used to confirm the diagnosis in cases of doubt or abnormal radiological findings. In a previous study, molecular testing, miRNA extraction, cDNA synthesis, amplification, and relative quantification, besides statistical analysis, results, and discussion, were mentioned in all groups^27^.

### Parameter description

**Figure 1:**
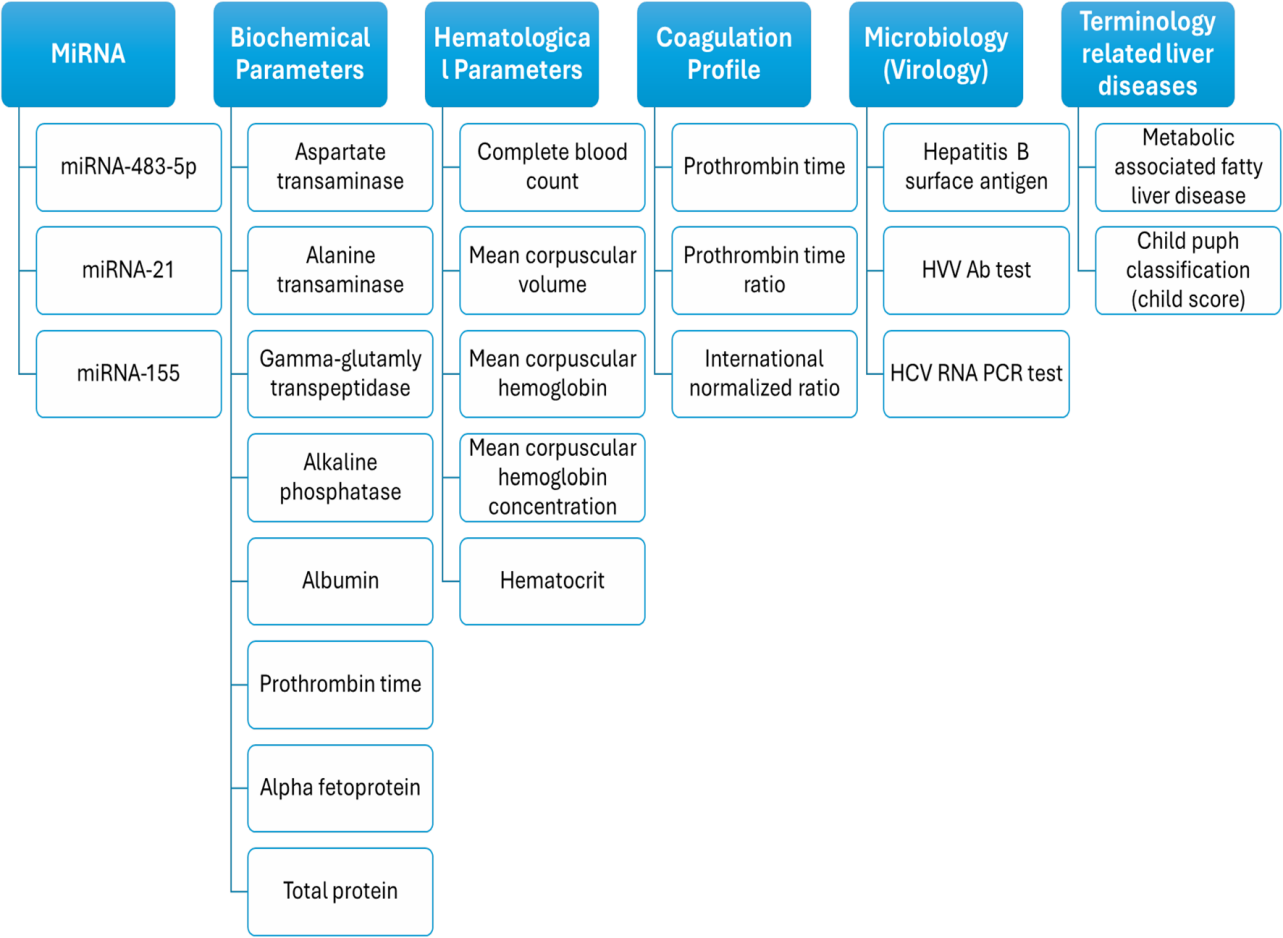
Different parameters and attributes for HCC studies.

The categories of all parameters related to the three studies are summarized in Fig. 1. These parameters include miRNA, biochemical, hematological, coagulation profile, microbiology parameters, and other parameters related to liver disease. Table 1 shows the parameters with their description. It should be mentioned that all of these parameters are continuous numbers.

**Table 1:** Dataset features description.

Parameter	Description
AST	Aspartate transaminase (AST) is a liver enzyme
ALT	Alanine transaminase (ALT) is a liver enzyme
ALP	Alkaline phosphatase (ALP) is a liver enzyme
GGT	Gamma-glutamy transpeptidase (GGT) is a liver enzyme
T. BIL	Total Bilirubin (T.Bil) is the amount of bilirubin in your blood
D. Bil	Direct Bilirubin (D.Bil) measures the bilirubin that is made in your liver
Alb	Albumin (Alb) is a protein made by the liver
TP	Total Protein (TP) measures the amount of protein in your blood which synthesized by liver
UREA	It is used to measure the renal function of the liver
Creat	Creatinine (Creat) is used to measure the renal function of the liver
RBS	A random blood sugar (RBS) is one method for measuring the amount of glucose or sugar circulating in a person’s blood at any time of day. For a glucose tolerance test, normal ranges are typically 140 mg per deciliter or lower
Hb	Hb indicate the quantity of the hemoglobin in the blood
RBCs	RBCs indicates the amount of the red blood cells
WBCs	WBCs indicates the amount of the white blood cells
PLT	PLT is platelet count in the blood
HCT	Hematocrit (HCT) blood test measures the number of red blood cells (RBCs) you have in relation to white blood cells and platelets
MCV	Mean corpuscular volume (MCV) measures the average size of the red blood cells
MCH	Mean corpuscular hemoglobin (MCH) is the average quantity of hemoglobin in a red blood cell
MCHC	Mean corpuscular hemoglobin concentration (MCHC) test is a standard part of the complete blood count (CBC) that is done during blood analysis, and the MCHC value is used to evaluate the severity and cause of anemia
Pt	Prothrombin time (Pt) is a protein made in liver where It helps blood to clot. A prothrombin time test measures how long it takes time for blood to clot. Albumin and Pt measure the synthetic function of the liver
1/Pt	The prothrombin time ratio is the ratio of a subject's measured prothrombin time (in seconds) to the normal laboratory reference PT
Conc	It is present at a concentration of approximately 100 μg/ml in normal plasma. Prothrombin (factor II) is a vitamin K–dependent coagulation factor. On activation, prothrombin is proteolytically cleaved to form thrombin, and in turn acts as a serine protease that converts fibrinogen to fibrin
INR	The international normalized ratio (INR) is the ratio of a patient's prothrombin time to a normal (control) sample, raised to the power of the ISI value for the analytical system being used
AFP	AFP is an oncofetal protein produced during intrauterine life and decline after birth to reach a very low serum levels in adults (normal range 0–20 ng/ml in adult serum)
MiRNA	MicroRNAs diagnostic and prognostic markers for HCC. Each study has different miRNAs (e.g., miRNA-483-5p, miRNA-21, and miRNA-155) in blood which used to detect a signature of miRNAs in HCC patients

**(A) Biochemical parameters:** include liver and renal function tests where liver function tests (LFTs) are used to help diagnose and monitor liver disease or damage, they check the levels of certain enzymes; (aspartate transaminase (AST), alanine transaminase (ALT), gamma-glutamyl-transpeptidase (GGT), and alkaline phosphatase (ALP)) and proteins (total protein (TP) and albumin (Alb)) in your blood. LFTs also include measuring serum bilirubin; total bilirubin (T. Bil) and direct bilirubin (D. Bil), a waste product that forms when red blood cells break down and is used to measure the conjugating function of the liver. Levels of LFTs that are higher or lower than normal can indicate liver problems. AFP level is increased in HCC and used as a tumor marker, but with low sensitivity and specificity, it is also used as a prognostic marker for follow-up of HCC patients. So, researchers try finding more specific markers for HCC than AFP. As well as, renal function is expressed by urea and creatinine levels.

**(B) Hematological parameters**

***(B-1) Complete Blood Count (CBC)*** includes hemoglobin, red blood cell count (RBCs), Mean Corpuscular Volume (MCV), Mean Corpuscular Hemoglobin (MCH), The mean Corpuscular Hemoglobin Concentration (MCHC), white blood cell count (WBCs), and platelet count (PLT). The normal ranges for a complete blood count are shown in Table 2.

**Table 2:** The normal ranges for a complete blood count.

Hemoglobin normal range	Male (ages 15+): 13.0–17.0 g/dL, female (ages 15 +): 11.5–15.5 g/dL
Hematocrit normal range	Male: 40–55%, female: 36–48%
Platelet count normal range	Adult: 150,000–400,000/mL
White blood cell (WBC)	Adult: 5000–10,000/mL

**(C) Coagulation profile**

***(C-1) Prothrombin time (PT)****:* The liver contains the protein prothrombin, which aids in blood clotting. A PT test calculates the time it takes for blood to clot. The liver's capacity for synthesis is measured by albumin and PT. Assays assessing the extrinsic route and common coagulation pathway are included with their derived measurements of prothrombin ratio (PR) and international normalised ratio (INR). Protime INR and PT/INR are other names for this blood test. They are applied in measuring warfarin dose, liver damage, and vitamin K status to ascertain the blood's propensity to clot. Fibrinogen, prothrombin, proaccelerin, proconvertin, and X are the coagulation factors that the PT tests for (Stuart–Prower factor). The analytical approach determines the reference range for PT, which is typically between 12 and 13 s, and the INR is 0.8–1.2.

***(C-2) Prothrombin time ratio (PT%)****:* The prothrombin time ratio measures the difference between a subject's measured prothrombin time (measured in seconds) and the standard laboratory reference Pt. The INR has taken the role of the Pt ratio, which changes depending on the particular chemicals employed.

***(C-3) International normalized ratio (INR)****:* Depending on the analytical technique used, a prothrombin time done on a healthy individual will have a different result (in seconds). This is brought on by changes in the manufacturer's tissue factor utilised in the reagent to conduct the test across various kinds and batches. To standardise the findings, the INR was developed. For every tissue factor, each manufacturer gives an international sensitivity index value (ISI). A particular batch of tissue factors are compared to an international reference tissue factor using the ISI value. The ISI is typically 2.0–3.0 for less sensitive thromboplastins and 0.94–1.4 for more sensitive thromboplastins. The prothrombin time ratio between a patient and a healthy (control) sample, multiplied by the ISI value for the analytical equipment being utilized, yields the INR.

**(D) Microbiology parameters**

***(D-1) Hepatitis B surface antigen (HBsAg):*** is a blood test ordered to determine if someone is infected with the hepatitis B virus. Blood that tests positive for HbsAg indicates that the patient is a viral carrier who may spread the infection to others through blood or other bodily fluids.

***(D-2) HCV Ab test****:* is used for initial screening for hepatitis C, which detects the presence of hepatitis C antibodies in serum. The result of the test is reported as negative or positive.

***(D-3) HCV RNA PCR test****:* is used to determine whether the hepatitis C virus (HCV) exists in your bloodstream. The test can determine exactly how much virus is in your blood if it is present. The viral load refers to the quantity of viruses present in your blood.

**(E) Parameters related to liver diseases**

***(E-1) Metabolic-associated fatty liver disease (MAFLD)****:* non-alcoholic fatty liver disease (NAFLD), also known as non-alcoholic steatohepatitis, is the main cause of liver disease worldwide and is quickly becoming the most prevalent cause of liver transplantation. The new change in terminology to MAFLD refocuses the conceptualization of this disease entity on its metabolic roots. It may spark a paradigm shift in its therapy, notably in the context of liver transplantation.

***(E-2) Child–Pugh Classification (child score):*** A common rating method for the severity of liver failure in cirrhotic individuals is the Child–Pugh classification. When adult patients have portosystemic shunting operations, the Child–Pugh class (A, B, or C) has historically been employed as a prognostic marker for operational death rate. For patients with Child–Pugh class B, the estimated 1- and 5-year survival rates are 95% and 75%, respectively, while for patients with Child–Pugh class C, the estimates are 85% and 50%. This method measures ascites, encephalopathy, serum albumin, bilirubin, and PT, among other things. The Child–Pugh scoring method then assigns points to various levels of each characteristic, and grades are subsequently determined using the sum of the points.

### Ethics approval and consent to participate

All methods were carried out in accordance with relevant guidelines and regulations. The three studies were approved by the Institutional Review Board of the National Liver Institute (NLI) Hospital and written informed consent was obtained from all participants**.**

## African vulture optimization algorithm

Recently, several nature-inspired metaheuristic algorithms have been proposed. They seek to strike a balance between exploitation and exploration, regardless of their variations in inspiration and search strategies^28^. At the moment, metaheuristic algorithms benefit from the beginning of the generation. To find new solutions, go through the exploration process. It gradually evolves into exploitation, emphasizing improving the accuracy of the solutions obtained during the research phase. Based on the best alternative previously offered by the population, the exploitation process develops a new solution^29^. Metaheuristic algorithms, therefore, depend on two essential elements to avoid being stuck in the local optimum. The majority of suggested metaheuristic algorithms are based on foraging and hunting strategies that occur naturally, however in this research, an alternative algorithm that is based on the habits of African vultures is employed^30^. The next subsection shows the inspiration analysis of the African vulture’s optimization (AVO) algorithm followed by the basic mathematical model for that analysis proposed by Abdollahzadeh et al.^30^.

### Inspiration analysis

Vultures may be found all over Africa, most of which follow a similar lifestyle. Vultures, especially in the tropics, are useful animals for preventing stinging and infecting carcasses. Vultures are an important part of the ecological systems theory, and their extinction poses several serious health hazards to humans. The African vultures can be categorized into three types: Ruppell's, white-backed, and Lappet-faced vultures^31^. The main difference between the three categories is their abler to obtain food. Compared to other vultures, the Lappet-faced Vulture has a better probability of finding food.

Vultures in the wild must travel large distances to get food. According to^31^, rotational flying is one of the vultures' most known types of flight. Vultures go to seek one species of vulture that has found food, and multiple species of vultures may move to a single food source, and these vultures will come into conflict with one another to gain food. Weak vultures encircle healthy vultures and feed by exhausting the stronger vultures, while starved vultures become more hostile^32^. This research on detecting and feeding various vultures in Africa was motivated by a new metaheuristic algorithm, which will be discussed furtherly.

### Mathematical model

In 2021, a new metaheuristic algorithm called AVOA based on African vulture behavior was proposed by Abdollahzadeh et al.^30^. In AVOA, African vultures' living habits and foraging behavior are simulated using the following criteria.

1. In the African vulture population, there are *N* vultures, and the size of *N* is determined by the algorithm based on the current scenario. Each vulture's position space has D dimensions, and the problem dimension determines the size of *D*. Similarly, depending on the complexity of the problem to be solved, a maximum number of iterations *T* must be determined in advance, indicating the maximum number of vulture actions.
2. The population of African vultures is divided into three groups based on their habits. The first group is to discover the best feasible solution among all vultures if the fitness value of the feasible solution is used to measure the quality position of the vultures. According to the second group, the practicable solution is the second best among all vultures. Aside from the two vulture groups mentioned above, the remaining vultures are separated into a third group.
3. The vulture's preferred foraging method is to go through the entire population. As a result, various vulture species have diverse functions in the community.
4. All vultures in AOVA aim to come close to the best vultures while avoiding the worst vultures.

In the exploration phase of the original AVOA, each vulture ${{\text{Y}}}_{{\text{i}}}$ can inspect different random areas depending on two alternative techniques, and a parameter called *R1* is utilized to select either strategy. This option must be set before the search operation and should have a value between 0 and 1, indicating how each of the two techniques is employed. If the value is more than or equal to the *R1* parameter, Eq. (1) is utilized. However, if rand *R1* is less than the parameter *R1*, Eq. (4) is utilized. In this situation, each vulture scans the environment at random for food.

1 $${{\text{Y}}}_{{\text{i}}+1}={{\text{P}}}_{{\text{i}}}-|2\times {\text{r}}\times {{\text{P}}}_{{\text{i}}}-{{\text{Y}}}_{{\text{i}}}|\times {\text{F}}$$

2 $${\text{F}}=(2\times {\text{r}}+1)\times {\text{L}}\times (1-\frac{{\text{i}}}{{{\text{T}}}_{{\text{Max}}}})$$

3 $${{\text{P}}}_{{\text{i}}}=\frac{{{\text{F}}}_{{\text{i}}}}{\sum_{{\text{i}}=1}^{{\text{N}}}{{\text{F}}}_{{\text{i}}}}$$

where ${\text{F}}$ indicates the vulture satiation rate, ${{\text{P}}}_{{\text{i}}}$ is the best vulture position, ${\text{r}}$ is a random number generated between zero and one, ${\text{t}}$ is the iteration number, ${\text{L}}$ is a random number generated between − 1 and 1, and ${{\text{T}}}_{{\text{Max}}}$ is the maximum number of iterations.

4 $${{\text{Y}}}_{{\text{i}}+1}={{\text{P}}}_{{\text{i}}}-{\text{F}}+{\text{r}}\times (({{\text{u}}}_{{\text{b}}}-{{\text{l}}}_{{\text{b}}})\times {\text{r}}+{{\text{l}}}_{{\text{b}}})$$

where ${{\text{l}}}_{{\text{b}}}$ is the lower boundary of the variables and ${{\text{u}}}_{{\text{b}}}$ is the upper boundary of the variables.

In the exploitation phase of the AVOVA, two different strategies are used. These strategies are siege-fight and rotating fight. Each vulture updates its position according to Eq. (5).

5 $${{\text{Y}}}_{{\text{i}}+1}=\left\{\begin{array}{c}\begin{array}{cc}{\text{Equ}}. (6)& |{\text{F}}|\ge 0.5\\ {\text{Equ}}. (9) & {\text{o}}.{\text{w}}\end{array}\end{array}\right.$$

6 $${{\text{Y}}}_{{\text{i}}+1}=\left\{\begin{array}{l}\begin{array}{cc}|2\times {\text{r}}\times {{\text{P}}}_{{\text{i}}}-{{\text{Y}}}_{{\text{i}}}|\times ({\text{F}}+{\text{r}})-({{\text{P}}}_{{\text{i}}}-{{\text{Y}}}_{{\text{i}}})& {\text{R}}2\ge {\text{r}}\\ {{\text{P}}}_{{\text{i}}}-({\text{T}}1+{\text{T}}2) & {\text{o}}.{\text{w}}\end{array}\end{array}\right.$$

7 $${\text{T}}1={{\text{P}}}_{{\text{i}}}\times (\frac{{\text{r}}\times {{\text{Y}}}_{{\text{i}}}}{2\uppi })\times {\text{cos}}({{\text{Y}}}_{{\text{i}}})$$

8 $${\text{T}}2={{\text{P}}}_{{\text{i}}}\times (\frac{{\text{r}}\times {{\text{Y}}}_{{\text{i}}}}{2\uppi })\times {\text{sin}}({{\text{Y}}}_{{\text{i}}})$$

where ${\text{R}}2$ is a random parameter generated between zero and one, and ${{\text{P}}}_{{\text{i}}}$ indicates one of the two best vultures in the current iteration's position vector.

9 $${{\text{Y}}}_{{\text{i}}+1}=\left\{\begin{array}{l}\begin{array}{ll}\frac{{\text{S}}1+{\text{S}}2}{2} & {\text{R}}3\ge {\text{r}}\\ {{\text{P}}}_{{\text{i}}}-({{\text{P}}}_{{\text{i}}}-{{\text{Y}}}_{{\text{i}}})\times {\text{F}}\times {\text{Levy}}({{\text{P}}}_{{\text{i}}}-{{\text{Y}}}_{{\text{i}}}) & {\text{o}}.{\text{w}}\end{array}\end{array}\right.$$

10 $${\text{S}}1={{\text{Y}}}_{1,{\text{i}}}^{*}-\frac{{{\text{Y}}}_{1,{\text{i}}}^{*}\times {{\text{Y}}}_{{\text{i}}}}{{{\text{Y}}}_{1,{\text{i}}}^{*}\times {{{\text{Y}}}_{{\text{i}}}}^{2}}\times {\text{F}}$$

11 $${\text{S}}2={{\text{Y}}}_{2,{\text{i}}}^{*}-\frac{{{\text{Y}}}_{2,{\text{i}}}^{*}\times {{\text{Y}}}_{{\text{i}}}}{{{\text{Y}}}_{2,{\text{i}}}^{*}\times {{{\text{Y}}}_{{\text{i}}}}^{2}}\times \mathrm{F }$$

where ${\text{R}}$3 is a random parameter generated between zero and one. In the present iteration, ${{\text{Y}}}_{1,{\text{i}}}^{*}$ and ${{\text{Y}}}_{2,{\text{i}}}^{*}$ are the best vulture of the first group and the second group, respectively. ${\text{Levy}}$ is the levy flight method is used to improve the exploration abilities of VOA.

## The proposed HCC detection model

**Figure 2:**
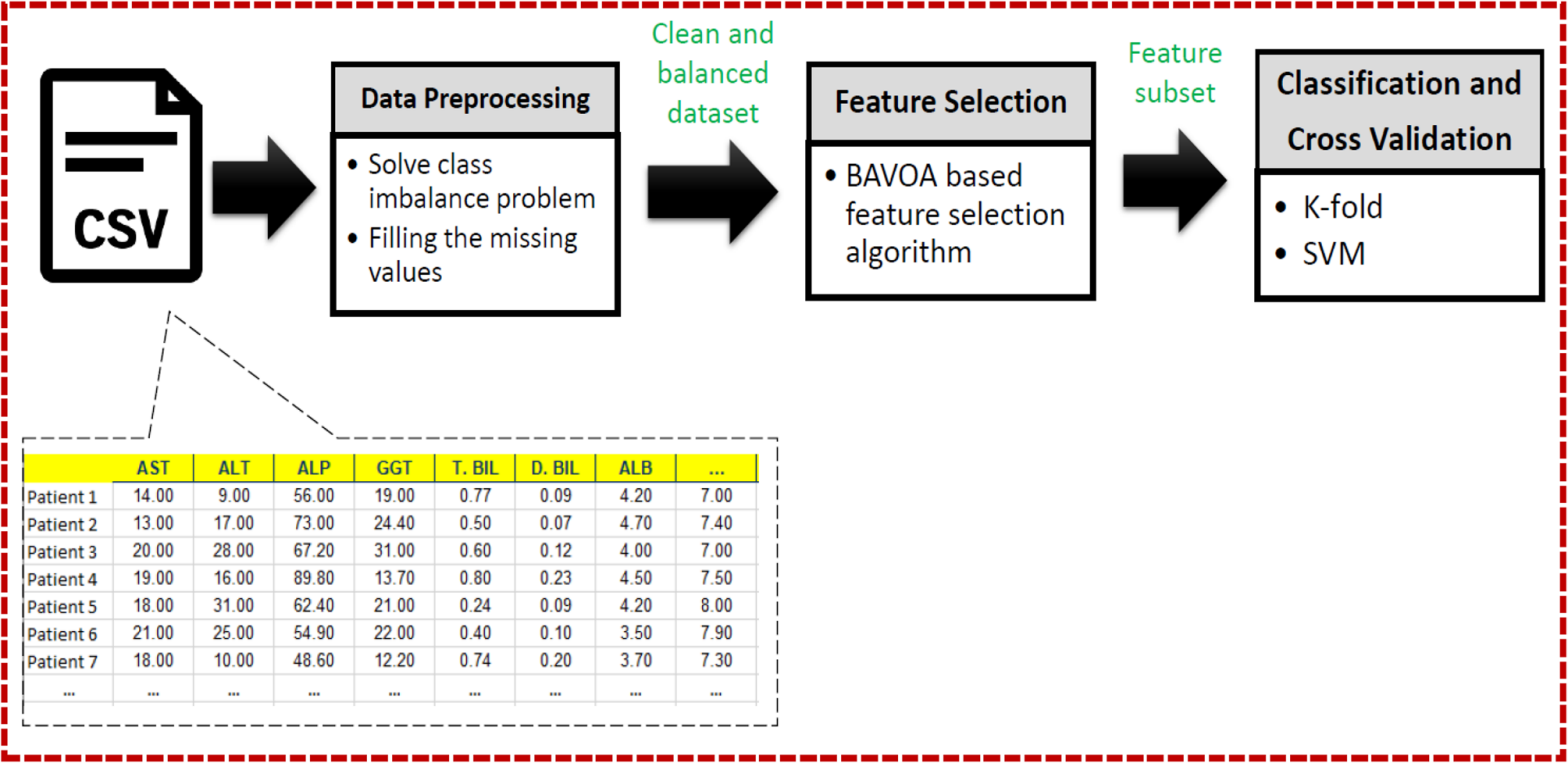
The block-diagram of the proposed HCC detection model.

The proposed model aims to evaluate the diagnostic and prognostic potential of circulating miRNAs in HCC patients and correlate them to the clinical and pathological parameters of the patients using three studies of HCC in Egyptian patients. It also can be considered as an early detection model of HCCcancer based on miRNAs biomarkers. The proposed model consists of three main phases: data preprocessing phase, feature selection based on the proposed BAVO algorithm phase, and finally, classification as well as cross-validation phase. The first phase namely the data preprocessing phase introduces the main problems associated with the collected real datasets. Additionally, in this phase, a solution for each problem is proposed. In the feature selection based on the proposed BAVO algorithm phase, a new binary version of the BAVO swarm-based algorithm is introduced. Finally, in the last phase, namely the classification and cross-validation phase, the performance of the overall proposed HCC detection model is evaluated using different evaluation criteria such as accuracy, sensitivity, precision, f-score, and specificity, where the support vector machine is utilized. Moreover, to prove the reliability of the proposed model, the k-folds cross-validation method is adopted. Figure 2 shows the architecture of the overall proposed HCCcancer detection model. A detailed description of each phase is presented in the following subsections.

### Data preprocessing phase

In the current work, the adopted dataset for each case study suffers from two main problems. These problems are imbalanced datasets and missing values problems. Therefore, three well-known and recent sampling techniques were employed to tackle the imbalanced classes’ distribution problem for the adopted datasets. These techniques are the SMOTE^33^, the SASYNO technique^34^, and the RUS method.

**Figure 3:**
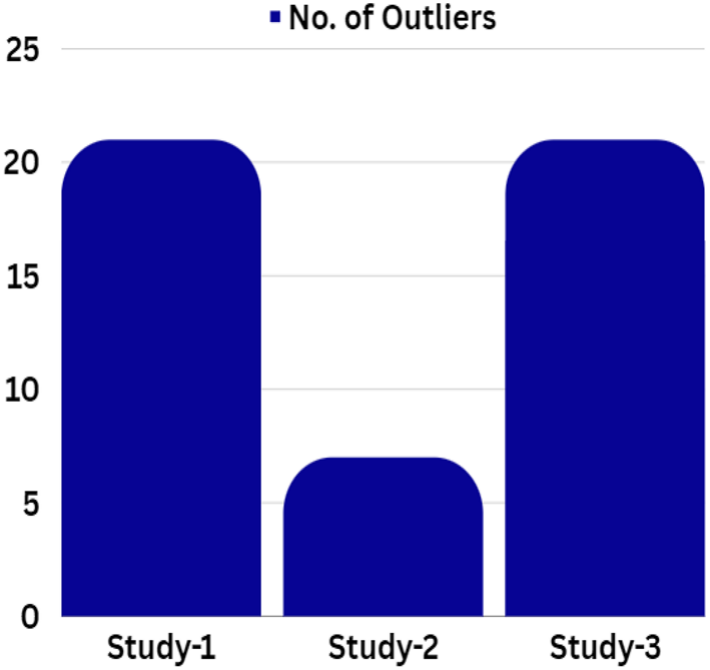
Number of outliers for the adopted datasets for each study.

Figure 3 depicts the number of outliers within each dataset used in the three studies. This information is critical in deciding on an appropriate method for dealing with missing values, with the nature of the dataset playing a significant role in this decision. When determining the best method for dealing with missing values, the presence of outliers is critical. Outliers are data points that deviate significantly from the average.

The Z-scoring method is one of well-know methods to identify the outliers from the data. In this paper, it was employed with the outlier threshold greater than 3 or less than -3. This threshold value indicates the number of standard deviations away from the mean. Each data point for the three adopted datasets were evaluated for outliers using the z-scoring method. The total number of samples in the first study is 100 with number of outliers as can be seen from Fig. 3 equals to 21, while the total number of outlier samples in the second study is 7 out of 71 total number of samples. Finally, the total number of outlier samples in the second study is 21 out of 180 total number of samples. These outliers have the potential to distort measures of central tendency, such as the mean, which is heavily influenced by extreme values. Because of this influence, the mean method is less suitable for datasets with outliers. The median method, on the other hand, which represents the middle value when the data is sorted, is a reliable measure of central tendency. It is less sensitive to extreme values than the mean. As a result, median imputation is frequently preferred over mean imputation in datasets with outliers. The median provides a more reliable estimate of the data's center, making it a better choice for dealing with missing values. The maximum imputation method, on the other hand, which involves replacing missing values with the highest observed value in the dataset, may not be appropriate for continuous data because it can introduce artificial discontinuities and skew the distribution.

In this paper, any missing values are imputed by replacing them with the median value of the known data points for a certain feature within a given class. This method assures that the imputed values are indicative of the data's central tendency for that specific feature, taking into account the specific class to which the data point belongs. The median is a robust measure that is less sensitive to outliers, offering a more reliable estimate of the missing data. Equation (12) shows the mathematical definition of the adopted method, where *M*_*i*_^*t*^ is the missing value for a given *i*-th iteration and *t*-th dimension, and *C*_*r*_ is the median value of a class. It should be noted that the adopted datasets are a set of continuous numerical values only, and no categorical values exist.

12 $${\overline{M} }_{i}^{t}={median}_{i:{M}_{i}^{t}\in {C}_{r}}{M}_{i}^{t}$$

### Feature selection phase

In this phase, a new binary version of the African vulture optimization algorithm, the BAVO-based feature selection algorithm, is proposed. A detailed description of this algorithm is presented in the following subsections.

*Solution encoding*: given *N* features for the provided dataset, the position of each African vulture is encoded as a binary vector *Y* = (*y*_1_*, y*_2_*, …, y*_*N*_). Each bit in *Y* vector *y*_*D*_ ∈ 0*,*1*, D* = 1*,* 2*, …, N*. When *y*_*D*_ equals 0, the *D* − *th* feature isn't selected, while 1 means that *D*th feature is selected. In this paper, the position of each African vulture is guided to guarantee that the miRNA feature is involved during the optimization process, where the last element *y*_*N*_ is set to 1.

*Parameters initialization*: in the beginning, the algorithm starts with setting the constant parameters of the BAVO algorithm. These parameters are the maximum number of iterations *T*_*Max*_, the dimensionality size *D*, the searching boundary [*lb,ub*], the population size *N*, and the control parameters (*p*1*, p*2*, and p*3, Alpha, Beta, and Gamma). In this paper, *T*_*Max*_ set to 20, *D* respect to each case study, [*lb,ub*] to [0,1], *M* to 30, *p*1 to 0.6, *p*2 to 0.4, *p*3 to 0.6, Alpha to 0.8, Beta to 0.2, and Gamma to 2.5. The values of the control parameters are determined based on the trial and error method, where it was found that these parameters obtained the highest results.

*Fitness function*: This paper adopts a wrapper-based feature selection method. Thus the classification accuracy of a supervised ML algorithm is used to evaluate how far good an African vulture's position is. *K*-nearest neighbor, known as *K-NN,* is one of the well-known algorithms. This paper uses k-NN to ensure the goodness of the selected features, where *k* is set to 3 with minimum Euclidean distance. This is due to its implementation simplicity and low computational time compared with more complex ML algorithms such as Support Vector Machines (SVM) and neural networks. In this paper, each position of the African vulture represents a feature subset, where the fitness function is composed of two objectives: the classification accuracy and the number of the selected feature. The best candidate position is the one that maximizes the classification accuracy while minimizing the number of selected features. Equation (13) defined the used fitness function.

13 $$F\left({{\text{Y}}}_{{\text{i}}}\right)=\omega xACC\left({{\text{Y}}}_{{\text{i}}}\right)+\left(1-\omega \right)X\frac{\sum_{i=1}^{N}{{\text{Y}}}_{{\text{i}}}}{N}$$

where *Acc* (${{\text{Y}}}_{{\text{i}}}$) is the obtained classification accuracy from *K*-NN using the ${{\text{Y}}}_{{\text{i}}}$ feature subset, $\sum\nolimits_{i = 1}^{N} Y$ is the number of the selected features, *w* is the weight of the classification accuracy, and 1 − *w* is the weight of the percentage of the eliminated features. In this paper, *w* is set to 0.9 because classification accuracy is more critical in a feature selection algorithm. Additionally, the k-fold cross-validation method is used to avoid feature selection bias. Thus this paper used a threefold cross-validation method to determine the *Acc*(${{\text{Y}}}_{{\text{i}}}$) in Eq. (13).

*The updating positions*: at each iteration, the position of each African vulture is updated according to Eqs. (14) and (15). Since the search space must be in the binary format, the continuous values are converted to binary using a transfer function called the sigmoid function. The mathematical definition of this function is represented in the following equations.

14 $${Y}_{i+1}^{t}=\left\{\begin{array}{c}1 if(Sigmoid \left({Y}_{i+1}^{t}\right))\ge r\\ 0 if(Sigmoid \left({Y}_{i+1}^{t}\right))<r\end{array}\right.$$

15 $$Sigmoid\left( {Y_{i + 1}^{t} } \right) = \frac{1}{{1 + e^{{ - 10\left( {Y_{i}^{t + 1} - 0.5} \right)}} }}$$

where *r* is a random number in [0,1], *t* runs from 1 to *N*, and *i* indicates the iteration number and runs from 1 to *T*_*Max*_.

*The termination criteria*: the optimization process is repeated until it reaches a termination condition. This paper selects the termination criteria as the maximum number of iterations. When the algorithm reaches the last iteration, the best feature subset (the best position of the African vulture) with its best fitness value is reported. Algorithm 1 show the pseudo-code of the overall the proposed BAVO feature selection algorithm.
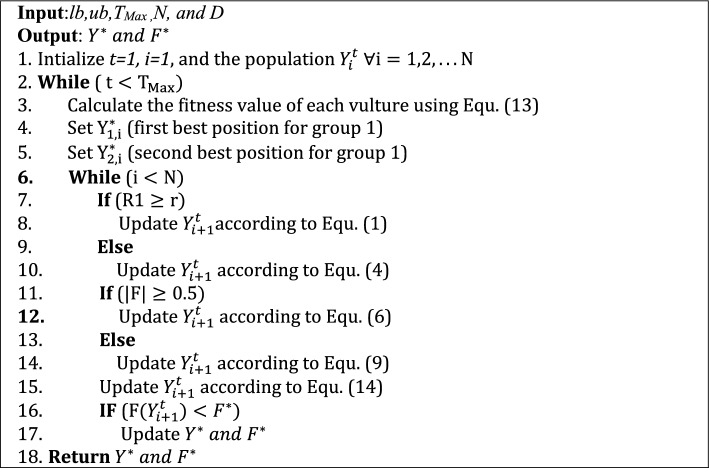


*The computational complexity*: the computational complexity of the proposed BAVO algorithms depends on three main phases; the Initialization of the vultures' positions, the fitness function, and the positions updating of vultures. The first phase, namely the initialization phase, takes *O* (*M*), while the calculation of the fitness function phase takes *O*(*t*_*Max*_ × *M* × *K*), where *K* is the folds number of the cross-validation method. Finally, the last phase, namely the positions updating of vultures phase, takes *Max*(*O*(*t*_*Max*_ × *M*) × *K, O*(*t*_*Max*_ × *N* × *M*)). So the overall computation time of the proposed BAVO is *Max*(*O*(*M* × (*t*_*Max*_ × *K, O*(*t*_*Max*_ × *N* × *M*)) or *O*(*M* × (*t*_*Max*_ × *K* + *t*_*Max*_*N*)).

### Classification and cross-validation phase

In this paper, SVM with the k-folds cross-validation method is adopted. SVM is one of the most used supervised ML algorithms, and it's used to solve classification and regression problems. SVM works by locating a hyperplane that categorizes the various classes. The difficulty here is determining which hyperplane to use to differentiate the classes, as there may be more than one hyperplane for a given problem as specified by margins.

In k-folds cross-validation, the whole data samples are randomly partitioned into approximately k equal-size subsets. For each k time, one subset is used for training, and another k − 1 subset is used for testing purposes. When the iteration reaches k, the average error rate is calculated to obtain a single estimation. In this paper, the value of k is set to 10.

## Results and analysis

The current study provides a complete analysis of the results for each phase of the proposed model for the three studies. The experimental results prove that the circulating miRNA-483-5p, 21, and 155 could be novel early diagnostic and prognostic biomarkers for detecting HCC. The main contributions of this work can be summarized as follows: (1) machine learning-based model is proposed to evaluate the diagnostic and prognostic potential (value) of circulating miRNAs in HCC patients and correlating them to the clinical and pathological parameters of the patients using three studies of HCC in Egyptian patients. (2) The proposed model provides an early detection model of hepatocellular carcinoma based on miRNA’s biomarkers. (3) Solving missing values problem and imbalanced distribution of the clinical report's data for the three studies, which provide a significant improvement in all accuracy measures of the proposed model compared to statistical analysis approaches. (4) A new binary version of the African vulture's optimization algorithm based on the lifestyles of African vultures is proposed as a feature selection algorithm. (5) Feature selection algorithm indicates a strong relationship between miRNA and the class feature which means that miRNA can be used as prognostic biomarkers for the detection of HCC. Another observation is that the effectiveness of miRNA on the class feature is higher than the effectiveness of the AFP feature on the class feature.

The results of the three main conducted experiments are described as follows; the first experiment as previously described aims to analyze the main characteristic of the introduced new markers, miRNAs. Also, it addresses the problems associated with the collected dataset of the three studies through two sub-experiments. The first sub-experiment shows the improvement in performance after solving the imbalanced classes’ distribution problem for the three case studies, while the second sub-experiment concerns the enhancement of the system accuracy after solving the missing values problem. The second experiment in 5.2 aims to evaluate the prognostic and diagnostic potential of circulating RQ OF485-5p Gene, RQ miRNA-21, and RQ miRNA-155, and find the optimal descriptors along with the previous markers. Finally, the overall performance of the proposed hepatocellular carcinoma detection model is evaluated and compared with the previous studies in the third experiment reported in “Classification and cross validation results”. A complete discussion of the reported results is reported in “Discussion”. All the conducted experiments are tested with an Intel Core i7 CPU, with 16 GB RAM, using Matlab R2020.

### Data pre-processing results

In this section, how pre-processing phase affects the overall accuracy of the proposed system is explored. All studies associated with this research have problems with missing values and imbalance distribution of the data among different classes. Using the insertion process for missing values enhances the overall accuracy of all studies as shown in Table 3. The second study was suffering from a lot of missing values which affect the overall system accuracy to be 42.86%. Solving this missing values problem increase the overall accuracy to 81.43%.

**Table 3:** The results before and after filling in the missing values in terms of accuracy.

	Study 1	Study 2	Study 3
Before	89	42.86	55.56
After	**90**	**81.43**	**60.56**

Significant values are in bold.

Table 4 compares three different resampling approaches used to overcome the the class imbalance distribution problem in terms of accuracy. SMOTE (Synthetic Minority Over-sampling Technique), SASYNO (Self-Adaptive Synthetic Over-sampling), and RUS (Random Under-sampling) are among these strategies. SMOTE creates synthetic samples for the minority class, thereby balancing class proportions. SASYNO generates synthetic samples using a self-adaptive technique, dynamically modifying the oversampling process based on the properties of the input. RUS, on the other hand, entails removing samples from the majority class at random in order to equalize class sizes. According to the results in Table 4, SASYNO consistently outperforms both SMOTE and RUS across the majority of the adopted datasets per study. This shows that SASYNO's adaptive nature gives a more personalized and effective solution to the imbalanced class problem.

**Table 4:** SMOTE vs. SASYNO vs. RUS in terms of accuracy.

	Study 1	Study 2	Study 3
SMOTE	94	**92.73**	61.62
SASYNO	**94.67**	92.22	**82.4**
RUS	87.14	86.43	79

Significant values are in bold.

**Figure 4:**
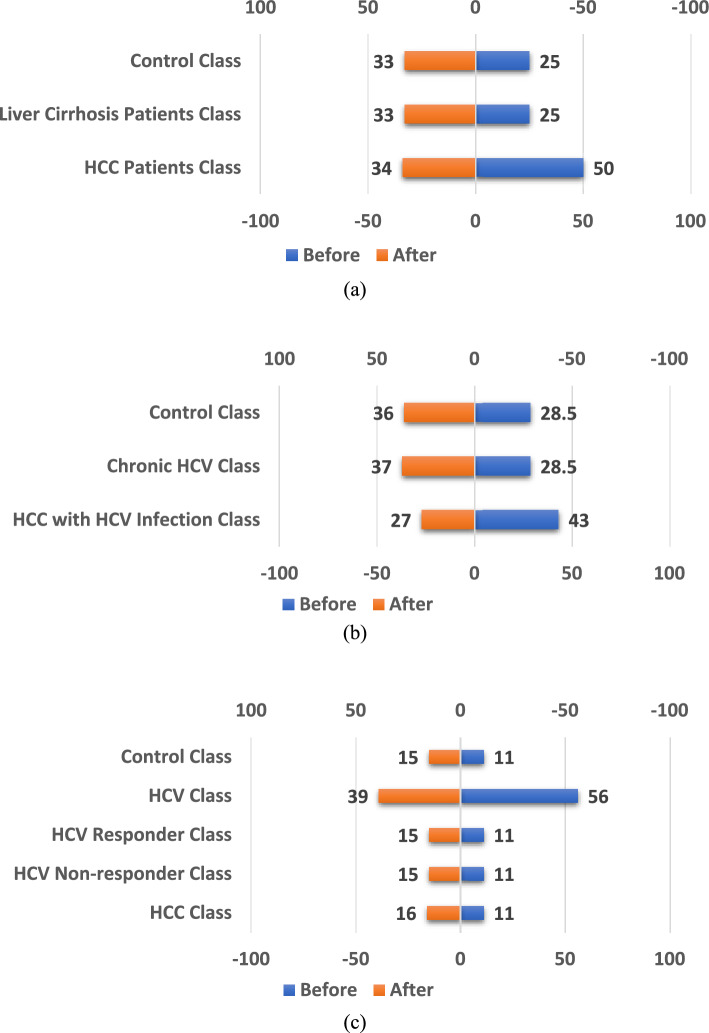
The classes’ distribution before and after using the SASYNO method; (**a**) the first study with miRNA 483-5p, (**b**) The second study with miRNA-21, and (**c**) the third study with miRNA-155.

It is worth reporting the effect of applying the SASYNO method on data samples in the three studies. This effect is shown in Fig. 4, where the class distribution in the percentage of each study is reported. As can be observed, applying an oversampling method can significantly adjust the number of samples in each class.

Table 5 shows the effect on classification results after using the SVM classifier with different kernel functions on data samples obtained from the SASYNO method. It can be seen that using the SASYNO method enhances the accuracy of all kernel functions for the three studies. The accuracy improvement for the first study ranges from 1% to 2.67%, while for the second study ranges from 3.65% to 7.786%, finally a huge improvement is achieved in the third study where it ranges from 25.18% to 37.64%. This is due to the dominance of the HCV class over the other classes in the third study compared to the other two studies.

**Table 5:** The obtained accuracy before and after applying the SASYNO method using different SVM kernel functions.

	Study 1		Study 2		Study 3	
	Before	After	Before	After	Before	After
RBF	90	92.67	87.14	91.11	40.56	78.2
Polynomial	**92**	**94.67**	**88.57**	**92.22**	**48.33**	**81**
Linear	**89**	**90**	**80**	**87.78**	**57.22**	**82.4**

Significant values are in bold.

### Feature selection results

**Figure 5:**
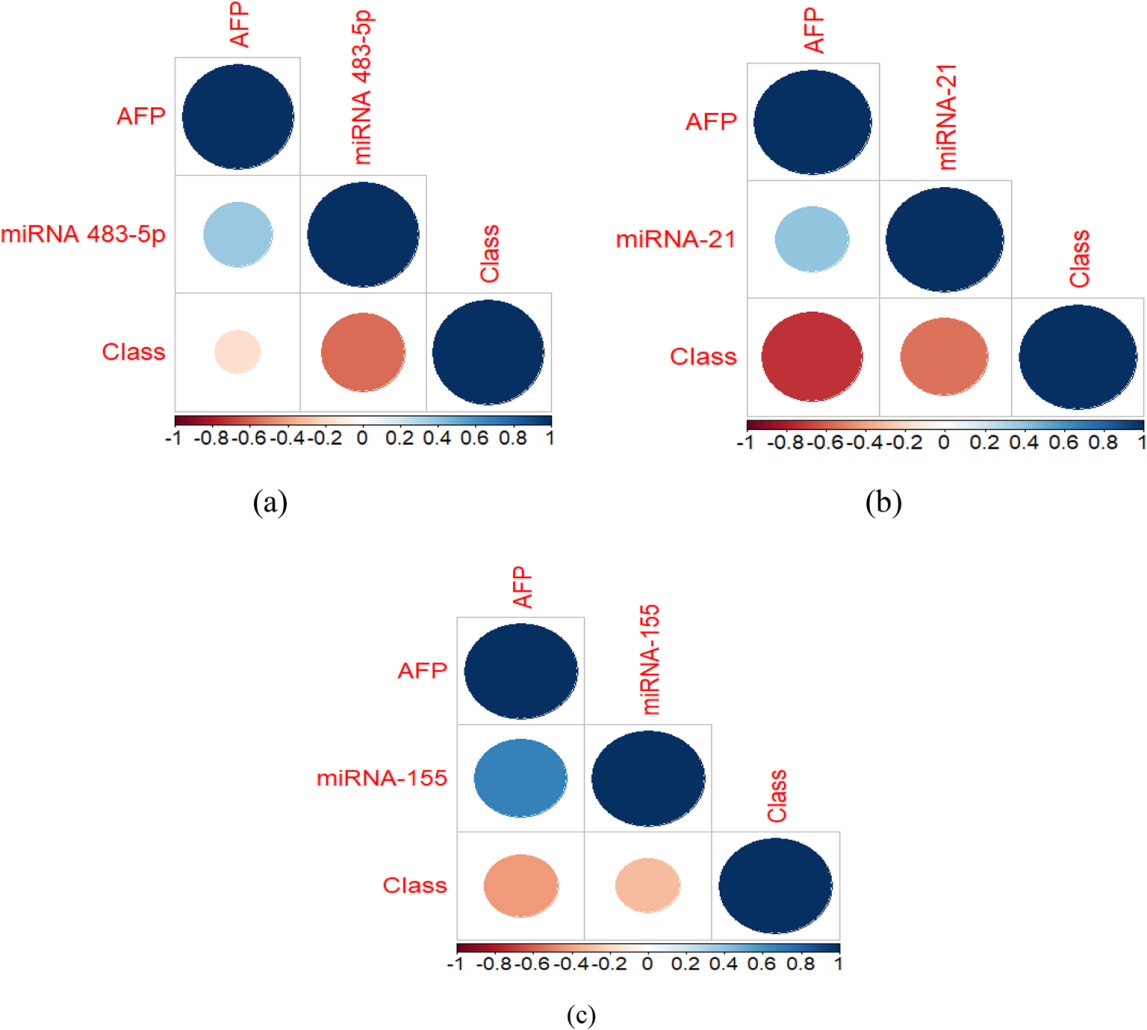
Correlation between features result; (**a**) the correlation of APF, miRNA, and the class for the first study miRNA 483-5p, (**b**) the correlation of APF, miRNA, and the class for the second study with miRNA-21, (**c**) the correlation of APF, miRNA, and the class for the third study with miRNA-155.

The main parameters that affect the diagnosis of HCC in Egyptian patients are reported. From Table 6, it can be observed that the proposed BAVO algorithm can optimally obtain the main parameters that affect and control classification results for the three studies. For further analyzing the characteristics of the adopted studies, the correlation between APF and miRNA in the first, second, and third studies are shown in Fig. 5. Correlation is one of the statistical measurements used to identify how close the relationship between two continuous features. Figure 5 shows that there is a strong relationship between miRNA and the class feature which means that miRNA can be used as prognostic biomarkers for the detection of HCC. Another observation is that the effectiveness of miRNA on the class feature is higher than the effectiveness of the alpha protein feature on the class feature. These results indicates that alpha protein does not appear in the best attributes that remarkably play an important role in the diagnostic and prognostic potential for HCC patients. Another observation is that the effectiveness of miRNA on the class feature is higher than the effectiveness of the AFP feature on the class feature.

**Table 6:** The best attributes obtained from the proposed BAVO-based feature selection algorithm.

Study 1	ALT, Creat, Hb, HCT, Conc, and RQ OF485-5p
Study 2	AST, GGT, Hb, RQ miRNA-21
Study 3	ALP, RQ miRNA-155

To analyze the impact of applying the proposed BAVO-based feature selection algorithm, the proposed HCC detection model is evaluated both before and after the use of a BAVO-based feature selection algorithm in Table 7. The results strongly demonstrate that the selection of relevant features is critical in improving the performance of the proposed model. The results, in particular, show a significant improvement in the model's performance in terms of accuracy, sensitivity, specificity, precision, and f1-score after incorporating relevant feature selection, emphasizing the relevance of this procedure in enhancing the model's efficacy.

**Table 7:** The performance of the proposed model with and without employing BAVO-based feature selection algorithm.

		Accuracy (%)	Sensitivity (%)	Specificity (%)	Precision (%)	F1-score (%)
Study-1	Before	**93.33**	**93.33**	**96.67**	**93.83**	**93.3**
	After	98	98	99	98.33	97.98
Study-2	Before	88.89	88.89	94.44	91.17	88.36
	After	**97.78**	**97.78**	**98.89**	**98.33**	**97.71**
Study-3	Before	78.4	78.4	94.6	77.65	76.99
	After	**83.2**	**83.2**	**95.8**	**85.05**	**79.23**

Significant values are in bold.

### Classification and cross validation results

In this subsection, the overall performance of the proposed model is evaluated using different measures such as overall accuracy, sensitivity, specificity, precision, and F1 score. These measures indicate how good is the proposed model by comparing the predicted results of the SVM classifier against the actual results reported in the original studies. The precision determines the count of models predicted correctly from all positive classes. A recall (also known as sensitivity) is the percentage of actual positive samples that are correctly predicted. The specificity of a test is called the true negative rate, which is the proportion of samples that test negative using the test in question that is a true negative. Finally, the F1-score is the harmonic average value of precision and recall, which refers to measuring how close precision and recall are. Additionally, two other evaluation metrics are considered. These metrics are Area Under the Curve (AUC) and Matthews Correlation Coefficient (MCC). AUC is used to differentiate between positive and negative classes. A greater AUC indicates better discrimination ability, with a perfect classifier reaching an AUC of 1. MCC considers true positives, true negatives, false positives, and false negatives, as opposed to accuracy, which can be misleading in imbalanced datasets. The MCC scales from − 1 to + 1, with + 1 being perfect prediction, 0 representing no better than random chance, and − 1 representing the entire disagreement between prediction and actual labeling. The obtained results from the SVM classifier after applying pre-processing phase and feature selection phase are conducted. These results are obtained after selecting optimal parameters using the BAVO algorithm and SASYNO method. Table 8 shows the accuracy, sensitivity, precision, f1-score, AUC, and MCC values for the three studies. As can be seen, the best results are achieved by the first study with an overall accuracy of 98%. The results also indicate the proposed model's great power in accurately diagnosing HCC. The significance of such accurate detection in the context of HCC stems from the opportunity for early diagnosis, which leads to timely intervention and improved treatment outcomes. Early diagnosis of HCC is crucial for implementing appropriate medical actions, perhaps improving the efficacy of treatment therapies, and eventually leading to a better prognosis for patients. The proposed model's accuracy in diagnosing HCC highlights its potential impact on advancing the field of liver cancer diagnosis.

**Table 8:** The obtained results from the proposed HCC liver detection model.

	Accuracy (%)	Sensitivity (%)	Specificity (%)	Precision (%)	F1-Score (%)	AUC	MCC
Study 1	98	98	99	98.33	97.98	0.9890	0.9625
Study 2	97.78	97.78	98.89	98.33	97.71	0.9963	0.9240
Study 3	83.2	83.2	95.8	85.05	79.23	0.7993	0.7847

Table 9 compares the performance of the proposed model with the state-of-the-art statistical analysis results for the three studies. As can be observed, the proposed HCC detection model based on using machine learning algorithms outperforms the traditional statistical analysis previously proposed in the literature in the majority of the studies. Also, it can be observed that machine learning can solve the problems within input data. Additionally, it can significantly select the best set of parameters that affect classification performance.

**Table 9:** Comparison with previous studies for the adopted three studies in terms of accuracy, sensitivity, and specificity.

		Accuracy (%)	Sensitivity (%)	Specificity (%)
Study-1	The proposed model	**98**	**98**	**99**
	^25^	90	88	92
Study-2	The proposed model	**97.78**	**97.78**	**98.89**
	^26^	93	90	92.5
Study-3	The proposed model	83.2	83.2	**95.8**
	^27^	**91.4**	**88.8**	92.4

Significant values are in bold.

It should be mentioned that the low performance of the proposed model in study two and study three is due to that these studies have incomplete columns or incomplete information despite the first study. In the second study, all Urea, Creat, RBS, HCT, MCV, MCH, MCHC, PT, 1/PT, and Conc values don't exist. Thus these columns are neglected in the experiment. However, we believe that some of these features/columns significantly affect the performance of the proposed model, as some of these already appeared on the optimal subset of features in the first study such as Creat and HCT. The same observation for the third study, where the values of Urea, Creat, RBS, RBCs, WBCs, HCT, MCV MCH, MCHC, PT, Conc., and INR are missing. Additionally, the feature column of GGT, D. BIL, and Hb has some missing values and these missing values are handled using the median method as previously described in the proposed model. However, with all of the missing values in the third study, the proposed model obtained better results in terms of accuracy, sensitivity, and specificity compared with the previously published results^27^.

## Discussion

Oncogenes and tumour suppressor genes are both dysregulated during the complicated aetiology of HCC^35–37^. By controlling tumour suppressor genes, miRNAs have been shown to be crucial in the development of tumours^38^. MiRNAs are non-coding, tiny endogenous RNA molecules that play a crucial part in a number of biological processes, such as cell differentiation, oncogenesis, and embryonic development^39^. By binding to the 3'-UTR of the mRNAs of certain target genes, they control gene expression at the post-transcriptional level, which in turn results in mRNA degradation or translational repression^14,40^. More than 50% of the human genome's miRNA genes are found in breakpoints, fragile sites, or locations linked to cancer, which are commonly implicated in chromosomal abnormalities such loss of heterozygosity, amplification, and breakpoints^41^. Additionally, miRNAs alter many cellular signalling pathways that are essential for cell survival, proliferation, and growth^42^. The development of new biomarkers for HCC diagnosis and/or prognosis is therefore necessary in order to better understand the molecular mechanisms behind HCC carcinogenesis. Due to their function, these markers may also serve as HCC treatment targets in the future. Therefore, several research looked at the expression levels of various miRNAs in HCC patients. Three separate studies are chosen from among these trials that were carried out on HCC patients at NLI, Menofia University. In order to assess the diagnostic and prognostic capabilities of these circulating miRNAs in patients with HCC and link them to the patients' clinical and pathological characteristics, three studies on three distinct miRNAs were conducted.

In the first study^25^, it was shown that the HCC group had a significantly higher level of miRNA483-5p than the liver cirrhotic group and control group (p < 0.001). However, no significant difference (p < 0.05) was found between the liver cirrhotic group and the control group. MiRNA-483-5 biomarker with AFP had (a sensitivity of 98% and specificity of 99%) as it was better than AFP alone (sensitivity = 88%, specificity = 92%).

For the second study^26^, the circulating miRNA-21 RQ levels showed a significant elevation in the early HCC stages (solitary focal lesion, absence of vascular invasion, tumor size < 3 cm, and TNM stages1 and 2) compared to the control group and CLD patients. At the same time, no significant changes were detected in serum AFP levels in the mentioned groups of that study^26^. ROC curve analysis of miRNA-21 as a marker for diagnosis of HCC revealed a sensitivity of 93% and specificity of 90% and accuracy of 92.5% compared to AFP which had a sensitivity of 75.2% and specificity of 92.3% and accuracy of 77% in such study^26^. Furthermore, using serum AFP and circulating miRNA-21 together improved the accuracy of HCC detection up to 97.7% while sensitivity was 97.7% and specificity 98.8%.

Also, the third study^27^ findings demonstrated that both the HCC group and the non-responder HCV group had high levels of miRNA-155 expression^27^. However, miRNA-155 level showed down expression in HCV responder group patients, this may return to the anti-inflammatory behavior of interferon and miRNA-155^43^. As miRNA-155 expression upregulates with disease development and down-regulating with healing. As well as this study revealed that miRNA-155 could be used as a detection biomarker for HCC with a sensitivity of 88.8% and specificity of 91%and accuracy of 91.4% compared to AFP which had a sensitivity of 76.2% and specificity of 87.3% and accuracy of 81.0%)^27^. Furthermore, using serum AFP and circulating miRNA-155 together to detect HCC cases provided an advantage over using them separately because the accuracy of combined use of both serum AFP and circulating miRNA-155 was 83.2% while sensitivity and specificity were 83.2% and 95.8%, respectively. This degradation in accuracy measures is due to the complete miss of parameters in the given study. The improvement of these accuracy measures was due to the ability of the proposed model to solve input data problems and select the optimal set of parameters that can effectively classify between HCC classes in different studies. Based on the proposed BAVO algorithm, miRNA-155, in combination with AFP, could be a unique diagnostic and prognostic biomarker for HCC identification and the possible therapeutic target for HCV and HCC infection.

However, some existing research papers have examined the early detection of HCC, each employing different biological data types, different nature of markers, and different models in their investigations. Additionally, only a few papers considered comparing their model with traditional methods (statistical-based methods). Meanwhile, not all of the existing papers reached an accuracy greater than 90% with proper justification. The following discussion will go further into a comparative examination of some of these papers in this domain.

The model proposed in^44^ utilizes machine learning-based computational algorithms to distinguish between cirrhosis tissues and HCC in patients who do not have HCC. In particular, numerical descriptors are extracted from gene expression profile datasets using the within-sample relative expression orderings approach. Through employing incremental feature selection combined with maximum redundancy and lowest relevance, an "11-gene-pair" has been identified. On the other hand, the model presented in this paper focuses on employing the BAVO-based feature selection algorithm to identify the best microRNA for the diagnosis and prognostic assessment of HCC in Egyptian patients. Unlike the machine learning model provided in^44^, which focuses on gene expression profiles, this paper emphasizes the selection of miRNAs as potential diagnostic and prognostic markers.

The model presented in^45^ used binary particle swarm optimization, t-test/ANOVA techniques, and machine learning algorithms for detecting HCC. The authors, however, did not address the issue of overfitting in their chosen dataset, which resulted in differences in classification findings. Significant differences were seen in the claimed classification accuracy between the training and testing sets for the 200 mRNAs and miRNAs that were chosen; these differences exceeded 19%. The training set attained a remarkable overall accuracy of 96.1%, while the testing accuracy declined to 76.9%. Unfortunately, the authors were unable to offer any explanation for this significant performance difference. Additionally, there was no comparison with traditional methods or state-of-the-art models, which limited the evaluation of the proposed model's effectiveness in a wider context of HCC identification.

The model proposed in^46^ focuses on identifying potential transcript biomarkers for early HCC prognosis utilizing RNA-Seq data and machine learning algorithms. This model analyzes RNA-Seq data from the healthy liver and distinct HCC cell types using five different machine-learning algorithms. The goal is to find transcriptome characteristics that distinguish healthy from HCC cell types. However, both models utilized machine learning algorithms to identify potential biomarkers for HCC and obtained higher detection results. However, they differ in the types of data collected, feature selection methods, and the nature of markers. The model in^46^ employed RNA-Seq data from healthy liver and several HCC cell models, but the data used in this paper includes hematological, biochemical, microbiological, and miRNA values obtained for each patient. The model in^46^ utilized recursive feature removal to acquire the fewest characteristics for discriminating between healthy and HCC cell models, whereas the proposed model employed the BAVO-based feature selection technique to identify optimum miRNAs as diagnostic markers of HCC. The model in^46^ focused on transcriptomic data to identify transcript biomarkers, while the proposed model in this paper includes a broader set of markers, such as those associated with liver disease such as miRNA, hematological, biochemical, and microbiological parameters.

While both models in the paper and the one proposed in^47^ utilized machine-learning algorithms and miRNAs as prognostic markers, they differ for the cancer type, the data content, and the utilized machine-learning algorithms. The proposed model in^47^ focuses on gastric cancer, while the proposed model in this paper addresses hepatocellular carcinoma. Additionally, the proposed model in^46^ used the TCGA database and focused on miRNA data, while the proposed model of this paper incorporates a broader range of markers associated with liver disease such as hematological, coagulation profile, biochemical, microbiological, and miRNA data.

The paper in^48^ focuses on reviewing the development of biosensors for the detection of microRNAs (miRNAs) as biomarkers for HCC, despite the objective of this paper. This paper is mainly focused on introducing an advanced approach based on employing machine-learning algorithms for HCC detection and the BAVO-based feature section algorithm for selecting optimal miRNAs as diagnostic markers.

However, both models, the proposed in^49^ and the proposed in this paper employed advanced computational techniques, these models differ in their specific used algorithms, data types, and focus areas. The model proposed in^49^ uses graph convolutional neural networks (GCN), and principle component analysis (PCA) for feature selection, while the proposed model in the paper uses machine-learning algorithms and BAVO for feature selection. The proposed model in^49^ used liver histopathology data of mice and gene expression levels, while the proposed in this paper uses different markers associated with liver disease of human patients. The main objective of the model in^49^ is to identify critical transitions during HCC development using liver histopathology data and gene expression levels, while the aim of this paper is to find the optimal miRNA as a diagnostic marker for HCC. Table 10 summarizes how the proposed model in this paper is similar to and different from other papers in the same area. As can be observed, each paper differs in terms of the utilized machine learning algorithm, the type of data, what they're studying, the biomarkers, and how precise their detections are.

**Table 10:** The similarities and dissimilarities between the proposed model in the paper and six other related papers in the domain.

References	Similarities	Dissimilarities
^44^	The main objective is to detect HCC in patients The authors utilizes ML algorithms The detection accuracy is good	It uses incremental feature selection algorithm combined with maximum redundancy and lowest relevance The employed feature selection algorithm is used to get the optimal gene expression profile
^45^	The main objective is to detect HCC in patients The authors utilizes swarm intelligence and ML algorithms	The detection accuracy is very low due to not handling the overfitting problem No comparison between either traditional methods or state-of-the-art models
^46^	The main objective is to early detection of HCC in patients The authors utilizes ML algorithms The detection accuracy is good	The authors employed RNA-Seq data from healthy liver and several HCC cells Recursive feature removal algorithm is employed to acquire the fewest characteristics for discriminating between healthy and HCC cell models
^47^	The authors utilized ML algorithms miRNAs are used as prognostic markers	The paper focuses on gastric cancer The authors adopted the TCGA database and focused on miRNA data
^48^	The paper focus on early detection of HCC	The paper focuses on reviewing the development of biosensors for the detection of miRNAs as biomarkers for HCC It is a review paper so no experimental results are considered
^49^	The authors utilized ML algorithms The detection accuracy is good	The authors employed GCN, and PCA for feature selection The authors adopted liver histopathology data of mice The main objective is to identify critical transitions during HCC development using liver histopathology data and gene expression levels

## Conclusion and future work

In Egypt, the rate of HCC has increased dramatically during the previous 10 years. It is classified as a heterogeneous sickness comprising a variety of neoplasms and unique changes in mRNA and miRNA expression profiles. Hepatitis C virus load and treatment response may be linked to miRNA- expression. An HCC detection model using ML algorithms is proposed to evaluate the diagnostic and prognostic potential (value) of circulating miRNAs in HCC patients and correlating them to the clinical and pathological parameters of the patients using three studies of HCC in Egyptian patients. The proposed model solved missing values and imbalanced data distribution in pre-processing phase. The optimal parameters that affect the classification and detection results are obtained using a novel BAVO. SVM with 10-fold cross-validation was used to detect different patient classes in the three studies based on selected parameters by BAVO. As shown in experimental results, the HCC detection model using ML algorithms superior to the traditional statistical-based method in terms of overall accuracy, sensitivity, and specificity. The improvement of these accuracy measures was due to the ability of the proposed model to solve input data problems and select the optimal set of parameters that can effectively classify between HCC classes in different studies. MiRNA-155 in conjunction with AFP may be a special diagnostic and prognostic biomarker for HCC detection as well as a potential therapeutic target for HCV and HCC infection, according to the suggested BAVO algorithm. A novel early diagnostic and prognostic biomarker for HCC detection may be miRNA-21 in the blood. Future cancer therapies may have a good case for using miRNA-21 expression interference. MiRNA-483-5p could be a unique HCC biomarker for distinguishing HCC patients from healthy persons. In addition, using serum AFP and circulating miRNA-483-5p together to detect HCC cases provided an advantage over using AFP alone. Because of the inherent limitation of a small sample size in this paper, further investigation involving a larger cohort of patients is necessary to confirm the robustness and generalizability of the proposed model's findings. Additionally, further investigations will include more diversified clinical sample, spanning various HCC stages and patient demographics. Moreover, more advanced machine learning algorithms will be further examined.

## Data Availability

Any data generated or analyzed during this study are available upon reasonable request. The corresponding author should be contacted if someone wants to request the data from this study. The dataset that adopted in this paper can be downloaded from https://data.mendeley.com/datasets/4x336ff4y2/1.

## References

[1] Wu Q, Li X, Long M, Xie X, Liu Q (2023). Integrated analysis of histone lysine lactylation (Kla)-specific genes suggests that NR6A1, OSBP2 and UNC119B are novel therapeutic targets for hepatocellular carcinoma. Sci. Rep..

[2] Wu S, Tang T, Zhou H, Huang J, Kang X, Zhang J (2023). LINC01343 targets miR-526b-5p to facilitate the development of hepatocellular carcinoma by upregulating ROBO1. Sci. Rep..

[3] Dhanasekaran R, Limaye A, Cabrera R (2012). Hepatocellular carcinoma: Current trends in worldwide epidemiology, risk factors, diagnosis, and therapeutics. Hepat Med..

[4] Wang Z, Qin H, Liu S, Sheng J, Zhang X (2023). Precision diagnosis of hepatocellular carcinoma. Chin. Med. J..

[5] Bose PP, Chatterjee U (2019). Advances in early diagnosis of hepatocellular carcinoma. Hepatoma Res..

[6] Wang W, Wei C (2020). Advances in the early diagnosis of hepatocellular carcinoma. Genes Dis..

[7] Kudo M, Kitano M, Sakurai T, Nishida N (2015). General rules for the clinical and pathological study of primary liver cancer, nationwide follow-up survey and clinical practice guidelines: The outstanding achievements of the liver cancer study group of Japan. Digest. Dis..

[8] Sakamoto M, Hirohashi S, Shimosato Y (1991). Early stages of multistep hepatocarcinogenesis: Adenomatous hyperplasia and early hepatocellular carcinoma. Hum. Pathol..

[9] Kojiro M, Wanless I, Alves V (2009). Pathologic diagnosis of early hepatocellular carcinoma: A report of the international consensus group for hepatocellular neoplasia. Hepatology.

[10] Theise, N. D., Park, Y. N., Curado, M. P., Sakamoto, M., Franceschi, S., Torbenson, M., Hytiroglou, P., Wee, A., & Kudo, M. WHO Classification of tumours of the Digestive System (4th ed., pp. 205–216) (International Agency for Research on Cancer, Lyon, 2010).

[11] Mao B, Xiao H, Zhang Z, Wang D, Wang G (2015). Microrna-21 regulates the expression of btg2 in hepg2 liver cancer cells. Mol. Med. Rep..

[12] Hu Q, Jiang H, Su J, Jiay Q (2013). Micrornas as biomarkers for hepatocellular carcinoma: A diagnostic meta-analysis. Clin. Lab..

[13] Valencia-Sanchez M, Liu J, Hannon G, Parker R (2006). Control of translation and mRNA degradation by miRNAs and siRNAs. Genes Dev..

[14] Ebert MS, Sharp PA (2012). Roles for microRNAs in conferring robustness to biological processes. Cell.

[15] Schetter AJ, Leung SY, Sohn JJ (2008). MicroRNA expression profiles associated with prognosis and therapeutic outcome in colon adenocarcinoma. JAMA.

[16] Zhu S, Si ML, Wu H, Mo YY (2007). MicroRNA-21 targets the tumor suppressor gene tropomyosin 1 (TPM1). J. Biol. Chem..

[17] Asangani IA, Rasheed SA, Nikolova DA, Leupold JH, Colburn NH, Post S, Allgayer H (2008). MicroRNA-21 (miR-21) post-transcriptionally downregulates tumor suppressor Pdcd4 and stimulates invasion, intravasation and metastasis in colorectal cancer. Oncogene.

[18] Lu Z, Liu M, Stribinskis V, Klinge CM, Ramos KS, Colburn NH, Li Y (2008). MicroRNA-21 promotes cell transformation by targeting the programmed cell death 4 gene. Oncogene.

[19] Zhu S, Wu H, Wu F, Nie D, Sheng S, Mo YY (2008). MicroRNA-21 targets tumor suppressor genes in invasion and metastasis. Cell Res..

[20] Datta J, Kutay H, Nasser MW, Nuovo GJ, Majumder S (2008). Methylation mediated silencing of microRNA-1 gene and its role in hepatocellular carcinogenesis. Cancer Res..

[21] Getzen E, Ungar L, Mowery D, Jiang X, Long Q (2023). Mining for equitable health: Assessing the impact of missing data in electronic health records. J. Biomed. Inf..

[22] Khoshgoftaar, T. M., Van Hulse, J., & Napolitano, A. Experimental perspectives on learning from imbalanced data. In *Proceedings of the 24th International Conference on Machine Learning*, 935–942 (2007).

[23] Murphy, P.M., Ali, K.M., Hume, T.M., Pazzani, C., Merz, K., & Brunk, C. Reducing misclassification costs. In *Eleventh International Conference on Machine Learning* (pp. 83–91) (Morgan Kaufmann, 1994).

[24] Prince M, Prathap PJ (2023). An imbalanced dataset and class overlapping classification model for big data. Comput. Syst. Sci. Eng..

[25] Maukar AL, Fathy W, Elbassal F, Farouk S, Azzam A, Elfert A, Soliman S, Elaskary S, Gomaa A (2018). Evaluation of circulating microRNA 483–5p as a useful diagnostic tool of hepatocellular carcinoma in Egyptian patients. Egypt. J. Med. Microbiol..

[26] El Gedawy G, Obada M, Kelani A, El-Said H, Ghanayem N (2017). Circulating miRNA-21 and programed cell death (PDCD) 4 gene expression in hepatocellular carcinoma (HCC) in Egyptian patients. Egypt. J. Med. Hum. Genet..

[27] Elfar W, Hanafy S, El-Said H, El-Fert A (2017). Study of miRNA-155 gene expression in Egyptian patients with chronic hepatitis C viral infection. Bull. Egypt. Soc. Physiol. Sci..

[28] Abualigah L, Diabat A, Mirjalili S, Abd Elaziz M, Gandomi AH (2021). The arithmetic optimization algorithm. Comput. Methods Appl. Mech. Eng..

[29] Abualigah L, Yousri D, Abd Elaziz M, Ewees AA, Al-Qaness MA, Gandomi AH (2021). Aquila optimizer: A novel meta-heuristic optimization algorithm. Comput. Ind. Eng..

[30] Soleimanian F, Mirjalili S, Abdollahzadeh B (2021). African vultures optimization algorithm: A new nature-inspired metaheuristic algorithm for global optimization problems. Comput. Ind. Eng..

[31] Houston DC (1974). The role of griffon vultures Gyps africanus and Gyps ruppellii as scavengers. J. Zool..

[32] Sarrazain F, Bose M (2007). Competitive behaviour and feeding rate in a reintroduced population of griffon vultures Gyps fulvus. Int. J. Avian Sci..

[33] Zhu T, Lin Y, Liu Y (2017). Synthetic minority oversampling technique for multiclass imbalance problems. Pattern Recogn..

[34] Gu X, Angelov P, Soares E (2020). A self-adaptive synthetic over-sampling technique for imbalanced classification. Int. J. Intell. Syst..

[35] Kajdasz A, Majer W, Kluzek K, Sobkowiak J, Milecki T, Derebecka N, Wesoly J (2021). Identification of RCC subtype-specific microRNAs: Meta-analysis of high-throughput RCC tumor microRNA expression data. Cancers.

[36] Malik J, Klammer M, Rolny V, Chan H, Piratvisuth T, Tanwandee T, Swiatek-de Lange M (2022). Comprehensive evaluation of microRNA as a biomarker for the diagnosis of hepatocellular carcinoma. World J. Gastroenterol..

[37] Piratvisuth T, Tanwandee T, Thongsawat S, Sukeepaisarnjaroen W, Esteban J, Bes M, Chan H (2022). Multimarker panels for detection of early stage hepatocellular carcinoma: A prospective, multicenter Case-Control Study. Hepatol. Commun..

[38] Uzuner, E., Ulu, G.T., Gürler, S.B., & Baran, Y. The Role of MiRNA in Cancer: Pathogenesis, Diagnosis, and Treatment. In: Allmer, J., Yousef, M. (eds) *miRNomics. Methods in Molecular Biology, *vol 2257 (Humana, New York, NY, 2022). 10.1007/978-1-0716-1170-8_18 34432288

[39] Peng B, Hu S, Jun Q, Luo D, Zhang X, Zhao H, Li D (2013). MicroRNA-200b targets CREB1 and suppresses cell growth in human malignant glioma. Mol. Cell. Biochem..

[40] Valencia-Sanchez MA, Liu J, Hannon GJ, Parker R (2006). Control of translation and mRNA degradation by miRNAs and siRNAs. Genes Dev..

[41] de Rooij LA, Mastebroek DJ, ten Voorde N, van der Wall E, van Diest PJ, Moelans CB (2022). The microRNA lifecycle in health and cancer. Cancers.

[42] Rojas-Pirela M, Andrade-Alviárez D, Medina L, Castillo C, Liempi A, Guerrero-Muñoz J, Kemmerling U (2022). MicroRNAs: Master regulators in host–parasitic protist interactions. Open Biol..

[43] Hu J, Huang S, Liu X, Zhang Y, Wei S, Hu X (2022). miR-155: An important role in inflammation response. J. Immunol. Res..

[44] Zhang ZM, Tan JX, Wang F, Dao FY, Zhang ZY, Lin H (2020). Early diagnosis of hepatocellular carcinoma using machine learning method. Front. Bioeng. Biotechnol..

[45] Hosseiniyan Khatibi SM, Najjarian F, Homaei Rad H, Ardalan M, Teshnehlab M, Zununi Vahed S, Pirmoradi S (2023). Key therapeutic targets implicated at the early stage of hepatocellular carcinoma identified through machine-learning approaches. Sci. Rep..

[46] Gupta R, Kleinjans J, Caiment F (2021). Identifying novel transcript biomarkers for hepatocellular carcinoma (HCC) using RNA-Seq datasets and machine learning. BMC Cancer.

[47] Azari H, Nazari E, Mohit R, Asadnia A, Maftooh M, Nassiri M, Avan A (2023). Machine learning algorithms reveal potential miRNAs biomarkers in gastric cancer. Sci. Rep..

[48] Lin X, Wang K, Luo C, Yang M, Wu J (2023). MicroRNA biosensors for early detection of hepatocellular carcinoma. Chemosensors.

[49] Han Y, Akhtar J, Liu G, Li C, Wang G (2023). Early warning and diagnosis of liver cancer based on dynamic network biomarker and deep learning. Comput. Struct. Biotechnol. J..

